# Increased Expression of Integrin-Linked Kinase Improves Cardiac Function and Decreases Mortality in Dilated Cardiomyopathy Model of Rats

**DOI:** 10.1371/journal.pone.0031279

**Published:** 2012-02-13

**Authors:** Rong Gu, Jian Bai, Lin Ling, Liang Ding, Na Zhang, Jiaxin Ye, Albert Ferro, Biao Xu

**Affiliations:** 1 Department of Cardiology, Drum Tower Hospital, Nanjing University Medical School, Nanjing, China; 2 Department of Clinical Pharmacology, Cardiovascular Division, King's College London, London, United Kingdom; Maastricht University, The Netherlands

## Abstract

**Aims:**

Integrin-linked kinase (ILK) is a multifunctional kinase linking the extracellular matrix to intracellular signaling pathways, whose activation in the heart gives rise to a number of functional consequences. The aim of this study is to demonstrate the therapeutic and survival benefit of cardiac ILK overexpression in a rat model of dilated cardiomyopathy.

**Methods and Results:**

The dilated cardiomyopathy model was generated in rats by intraperitoneal administration of six equal doses of doxorubicin over a 2 week period. Five weeks after the first injection, echocardiographic analysis demonstrated impaired cardiac function and, at that point, recombinant adenoviral vector harboring ILK cDNA or vehicle was injected into the myocardium, and the rats re-studied 4 weeks later. Compared with vehicle injection, ILK treatment ameliorated inflammatory cell infiltration and cardiomyocyte degeneration, as well as left ventricular dilation and dysfunction. ILK treatment was also associated with a reduction in apoptosis and an increase in proliferation of cardiomyocytes, as well as decreased oxidative stress and autophagic vacuole accumulation. Importantly, mortality was lower in rats following ILK treatment than in those following vehicle injection. In cultured neonatal rat cardiomyocytes, we also found that ILK overexpression protected against doxorubicin-induced apoptosis, giving rise to an increase in their proliferation.

**Conclusions:**

These data demonstrate for the first time that ILK gene therapy improves cardiac function and survival in a model of dilated cardiomyopathy, and this may be mediated through suppression of inflammation, prevention of ventricular remodeling, inhibition of cardiomyocyte apoptosis and autophagy, and stimulation of cardiomyocyte proliferation.

## Introduction

Despite important advances in medical as well as surgical and device treatment, chronic heart failure remains an important worldwide health problem with a poor prognosis [1]. Since it is characterized by loss of cardiomyocytes combined with impaired function of the remaining cells and often decreased blood flow (especially if the underlying etiology is ischemic heart disease), cell transplantation and gene therapy have been tried and have proved to be promising strategies. In particular, transplantation of mesenchymal stem cells [2], bone marrow mononuclear cells [3] and skeletal myoblasts [4], as well as gene therapy with hepatocyte growth factor [5], insulin-like growth factor [6] and vascular endothelial growth factor (VEGF) [7], have been demonstrated to improve cardiac function and to ameliorate many of the underlying pathophysiological features.

Integrin-linked kinase (ILK) is a widely expressed serine/threonine protein kinase and an important biomechanical sensor which becomes activated upon cell-matrix interaction, thereby exerting a variety of biological functions including induction of angiogenesis and regulation of cardiac contractility [8], ventricular hypertrophy [9], cell proliferation, survival and differentiation [10], [11]. Deletion of ILK from the murine heart results in dilated cardiomyopathy and spontaneous heart failure [12]. We have previously shown that ILK gene therapy can attenuate ventricular remodeling and improve cardiac function in a rat model of myocardial infarction [13]. Its beneficial effects may be mediated by increased angiogenesis and cardiomyocyte proliferation as well as reduced apoptosis. However, at present it is not known whether ILK gene therapy may improve cardiac function in models of heart failure and in the absence of underlying cardiac ischemia. The purpose of the present study was therefore to investigate whether overexpression of ILK can improve cardiac function of rats with doxorubicin-induced heart failure, a model of dilated cardiomyopathy, as well as to determine the mechanisms underlying its beneficial effects in this condition.

## Methods

### Ethics Statement

Animal experiments conformed to the Guide for the Care and Use of Laboratory Animals published by the US National Institutes of Health (NIH Publication No. 85-23, revised 1996) and was approved by the Ethics Review Board for Animal Studies of Nanjing Drum Tower Hospital (DTH ERBA 66.01/026D/2010).

### 3.1 Recombinant adenovirus construction

Recombinant adenoviral vector containing ILK and hrGFP cDNA (adeno-ILK) was constructed as previously described [13]. Experiments using adeno-ILK were performed in parallel with vector harboring neither of these (adeno-null).

### 3.2 Animal model of dilated cardiomyopathy

A model of dilated cardiomyopathy was generated in 220–250 g male Sprague-Dawley rats (n = 50) with six equal doses of intraperitoneal doxorubicin hydrochloride (Sigma; total dose 15 mg/kg) administered over 2 weeks as described previously [4]. Sham-treated rats (n = 6) were injected with the same volume of saline using the same procedure.

### 3.3 Echocardiography and hemodynamic assessment

Animals were examined 35 days (5 weeks) after the first doxorubicin injection, and again 28 days (4 weeks) after adenoviral vector delivery. After anesthesia using a mixture of intraperitoneal ketamine hydrochloride (50 mg/kg) and diazepam (5 mg/kg), rats were placed on a warm blanket, and echocardiographic long-axis and short-axis two-dimensional as well as M-mode tracings were obtained at the level of the papillary muscles (SONOS model 5500, Hewlett-Packard Co.) using an 8 MHz transducer. Left ventricular end-systolic diameter (LVESD) and end-diastolic diameter (LVEDD), as well as interventricular septal thickness in diastole (IVSd) and LV posterior wall thickness (LVPWT), were all measured by an observer blinded to the treatment, and averaged for three consecutive pulsation cycles. Percent left ventricular fractional shortening (%FS) was calculated as follows: %FS = (LVEDD-LVESD)/LVEDD×100 (%).

Hemodynamic indices (left ventricular systolic pressure (LVSP), left ventricular end-diastolic pressure (LVEDP), maximum positive and negative dP/dt (+dP/dt_max_, −dP/dt_max_), and heart rate (HR)) were obtained using a polyethylene catheter (PE 50, Becton-Dickinson) introduced into the left ventricle via the right carotid artery and connected to a pressure transducer (MPU-0.5, Nihon Kohden, Japan) following echocardiography.

### 3.4 Adenoviral vector delivery

35 days after the first injection, 40 out of 50 doxorubicin-treated rats (those who had survived and conformed to the heart failure diagnosis standard) were randomized to treatment with either adeno-ILK (n = 20) or adeno-null (n = 20). After induction of anesthesia as above, rats were intubated and ventilated (Jiangxi Teli, China) and, following incision in the left fourth intercostal space, 100 µl in total of adeno-ILK (corresponding to 3×10^9^ viral particles (VP)) or adeno-null (3×10^9^ VP) was injected into the left ventricle at 10 points, using a syringe attached to a 29-gauge needle as previously described [4]. Following this, the chest cavity, muscles and skin were sutured in 3 layers.

### 3.5 Tissue sample preparation

After hemodynamic assessment at 63 days (9 weeks), hearts were arrested in diastole by intravenous injection of 2 mol/L KCl. The myocardium was sliced into three blocks (basal, mid-region, and apical blocks) and the mid-region portion was cut into two further blocks, one of which was snap-frozen in liquid nitrogen and stored at −80°C for subsequent TUNEL examination, whilst the other was fixed in 4% neutral formalin for 24 h at room temperature followed by embedding in paraffin wax and cutting into 5 µm slices for subsequent histologic and immunohistochemical analysis. The remaining blocks were snap-frozen in liquid nitrogen and stored at −80°C for western blotting.

### 3.6 Histologic analysis

Paraffin-embeded slices (5 µm thickness) were stained with hematoxylin-eosin for morphologic examination or Masson's trichrome for interstitial fibrosis determination. Left ventricular diameter was measured at the level of the papillary muscle as described [14]. Left ventricular wall thickness, myofilament density, cardiomyocyte size and collagen volume fraction (CVF) were measured and analyzed using Image Pro Plus software (Media Cybernetics), and CVF was calculated as area of Masson's trichrome-stained connective tissue divided by total area of the image as described previously [15].

### 3.7 ILK expression and activity following adenoviral vector delivery

Frozen myocardium was homogenized using a Dounce tissue grinder in lysis buffer consisting of: 10 mmol/L Tris/HCl (pH 7.2), 150 mmol/L NaCl, 0.1% SDS, 1% sodium deoxycholate, 5 mmol/L EDTA, 50 mmol/L NaF, 1 mmol/L sodium orthovanadate, 1% (v/v) TritonX-100, protease inhibitor cocktail (Roche). Protein concentrations were quantified by BCA protein assay kit (Pierce). 50 µg protein per sample was separated by sodium dodecyl sulfate-polyacrylamide gel electrophoresis (SDS-PAGE), followed by transferring to polyvinylidene fluoride membranes (Immobilon-P, Millipore). After incubation with mouse anti-human ILK (1∶3000, BD Transduction Laboratories), rabbit anti-phospho-Akt (Ser473) (Cell Signaling Technology), rabbit anti-Akt (Cell Signaling Technology) or mouse anti-GAPDH (1∶3000, Kangchen Bio-Tech) antibodies, peroxidase-conjugated goat anti-mouse IgG or goat anti-rabbit IgG were used as secondary antibodies (1∶5000, Jackson ImmunoResearch). Bands were visualized by enhanced chemiluminescence detection system (Pierce Biotechnology Inc, Rockford, IL, USA). Results were presented as the ratio of band density values of ILK to GAPDH or ratio of phospho-Akt to Akt.

### 3.8 TUNEL assay

We detected apoptosis using DeadEnd Fluorimetric TUNEL System (Promega) according to the manufacturer's instructions. Frozen sections were counterstained with mouse monoclonal α-sarcomeric actin antibody (1∶50, Abcam) while Alexa Fluor 555 goat anti-mouse antibody (1∶250, Molecular Probes) was applied as secondary antibody. Cell nuclei were incubated with DAPI (Sigma), and then the sections were mounted and analyzed with a Fluoview 1000 confocal microscope (Olympus, Japan). The number of TUNEL-positive cardiomyocyte nuclei was manually determined. The total number of nuclei (exhibited as DAPI-positive signals) was automatically calculated using Image Pro Plus software (Media Cybernetics).

### 3.9 Immunohistochemical analysis of cardiomyocyte proliferation

Immunohistochemical analysis was performed using antibodies to phospho-histone H3 (1∶100 rabbit anti-phospho-histone H3; Cell Signaling Technology), Ki-67(1∶100 mouse anti-Ki-67, Zymed Laboratories) and CD45 (1∶50 mouse anti-CD45, Abcam). Briefly, paraffin-embedded sections were blocked for 30 min with 5% goat serum followed by incubation with primary antibodies overnight at 4°C. Detection and visualization were performed using the EnVision detection system (DAKO) after incubation with secondary antibody for 1 h at 37°C. The ratio of phospho-histone H3 or Ki67 positive nuclei to the total number of nuclei was determined and averaged for at least ten fields per sample.

### 3.10 TNF-α determination

Plasma TNF-α concentrations were determined using rat TNF-α ELISA kit (Bender MedSystems, Austria, Europe), according to the manufacturer's instructions.

### 3.11 Oxidative stress determination

We detected the concentration of MDA and activity of SOD in the plasma, both using commercially available kits (Nanjing Jiancheng Bioengineering Institute), according to the manufacturer's instructions.

### 3.12 Electron microscopy

Hearts were quickly and carefully dissected from sacrificed rats, portions of the left ventricle were cut into 1 mm^3^ cubes and fixed with 2% glutaraldehyde in 0.1 mol/L sodium phosphate buffer, pH 7.4, overnight. The fixed samples were then post-fixed with 1% OsO_4_, embedded, sectioned and analyzed by transmission electron microscopy.

### 3.13 Real-time polymerase chain reaction quantitation of beclin 1 mRNA

As previously described [16], total RNA was extracted from each sample using Trizol reagent (Invitrogen) and dissolved in DEPC-treated water. 2 µg of total RNA was reverse-transcribed into cDNA using the M-MLV reverse transcriptase (Invitrogen), according to the manufacturer's instructions. Real-time polymerase chain reaction was performed with a 7500 Real-Time PCR system (Applied Biosystems, CA, USA) using SYBR Premix Ex TaqTM (Takara Bio, Shiga, Japan). The primers for beclin 1 and GAPDH were as follows:

Beclin 1

S: TTCAATGCGACCTTCCATATCT

AS: TGATTTCATTCCATTCCACAGG

GAPDH

S: GGCAAGTTCAACGGCACAG

AS: GACGCCAGTAGACTCCACGAC

The reaction was performed at 95°C for 30 s, followed by 40 cycles of 95°C for 5 s and 60°C for 34 s. The dissociation stage was initiated at 95°C for 15 s, followed by 1 cycle of 60°C for 1 min and 95°C for 15 s. The relative gene expression for each sample was determined using the formula 2^−ΔΔCt^, to reflect target gene expression normalized to GAPDH levels.

### 3.14 Cardiomyocyte isolation and culture

Primary cultures of neonatal rat cardiomyocytes were prepared as previously described [17], with some modifications. Briefly, hearts were obtained from 1- to 2-day-old Sprague-Dawley rats. The hearts were washed with PBS, and the atria and aorta discarded. The ventricles were minced into 1–3 mm^3^ pieces, and then digested 4–5 times for 15 min each time with PBS containing 0.1% collagenase (type I, Sigma) and 0.1% trypsin (Sigma), at 37°C. The dissociated cells were collected by centrifugation and incubated in flasks (Corning) for 60 min at 37°C in a humidified atmosphere (5% CO2, 95% air), to allow attachment of non-cardiomyocytes. The non-adherent cardiomyocytes were harvested and seeded into six-well plates with coverslips. The myocytes were incubated in Dulbecco's modified Eagle's medium (DMEM) containing 10% FBS, 100 U/ml penicillin, and 100 µg/ml streptomycin. 5-Bromo-2′-deoxyuridine (BrdU; 0.1 mmol/L) was added to the culture medium for the first 48 h to inhibit proliferation of non-cardiomyocytes. Cardiomyocytes were then incubated in DMEM containing 0.5% FBS without BrdU. Using this method, we obtained cardiomyocytes at 90–95% purity, as determined by immunoflorescence staining with a monoclonal antibody against α-sarcomeric actin.

### 3.15 Adenovirus transfection of cultured cardiomyocytes

Cardiomyocytes were incubated with either adeno-ILK or adeno-null, 48 h after isolation, at a multiplicity of infection (MOI) of 100∶1, in DMEM containing 0.5% FBS. After 2 h, the media were removed and replaced with fresh media, and cells cultured for another 24 h before further experiments as below.

### 3.16 Cardiomyocyte viability assay

Cell viability was determined by MTT assay as previously described [18]. For this assay, the cardiomyocytes were seeded into 96-well plates with 2×10^4^ cells/well. 24 h after transfection, cardiomyocytes were treated with 1 µmol/L doxorubicin or vehicle for 24 h, followed by 0.5 mg/mL MTT for 30 min, at 37°C. They were then lysed in 100 µl DMSO. Absorbance was determined at 570 nm in an ELISA plate reader. All experiments were performed at least in triplicate, and results expressed as percentage of the control group.

### 3.17 Cardiomyocyte apoptosis assay

Cardiomyocytes were seeded into six-well plates with 3×10^5^ cells/well. 24 h following transfection, cells were treated with 1 µmol/L doxorubicin or vehicle for 24 h. The cardiomyocytes were then stained with propidium iodide (PI) and annexin V-APC according to the manufacturer's instructions (annexin V-APC apoptosis detection kit, Bender MedSystems, Vienna, Austria). Apoptotic cells were assessed for annexin V staining by gating on the fraction of PI-negative cells by flow cytometry (FACScan; Becton, Dickinson and Company, New Jersey, USA).

### 3.18 Determination of cardiomyocyte proliferation

96 h after transfection, the cardiomyocytes were fixed with 4% paraformaldehyde, rinsed with PBS, and permeabilized with 0.1% Triton X-100 in PBS. Endogenous peroxidase activity was quenched by incubating coverslips in 3% H_2_O_2_ for 10 min. After blocking with 1% bovine serum albumin , cells were incubated overnight with a polyclonal antibody against phosphohistone H3 (1∶100, Cell Signaling Technology), followed by detection and visualizion using the EnVision detection system (DAKO), according to the manufacturer's instructions. Phosphohistone H3-positive nuclei and total nuclei were counted per field at ×200 magnification. The ratio of phosphohistone H3-positive to total nuclei was calculated and averaged for at least ten fields per coverslip.

### 3.19 Statistical analysis

All results were expressed as mean ± SEM. Comparisons between groups were performed by unpaired Student's *t* test or ANOVA with Dunnett's post-test as appropriate. Survival was estimated from the date of adenoviral vector delivery until death or 4 weeks after adenoviral vector injection. The significance of differences in survival data was determined using Kaplan-Meier analysis. All data analysis was performed using SPSS 13.0 software. Statistical significance was defined as *P*<0.05 (two-tailed).

## Results

### 4.1 ILK gene therapy causes increased cardiac ILK expression and activity

**Figure 1:**
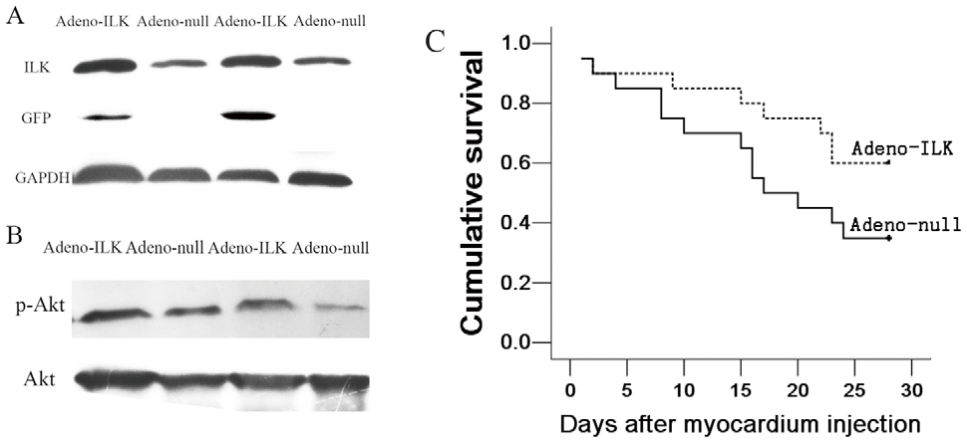
ILK gene therapy increases left ventricular ILK expression and activity, improves survival in doxorubicin-induced cardiomyopathy. A, Western blot showing elevated ILK protein expression in the left ventricular tissue of the adeno-ILK group as compared with the adeno-null group. The blot also shows green fluorescent protein (GFP) expression in adeno-ILK, but not adeno-null, treated hearts. Glyceraldehyde-3-phosphate dehydrogenase (GAPDH) expression is also shown as a housekeeping protein. B, Western blot showing left ventricular phospho-Akt (pAkt) and Akt expression. The ratio of pAkt to Akt was increased following adeno-ILK delivery relative to adeno-null control, in line with increased Akt phosphorylation due to elevated ILK activity. C, Kaplan-Meier survival curve for doxorubicin-treated rats treated with adeno-ILK (n = 20) and adeno-null (n = 20) (p = 0.093).

In a previous study [13], we investigated ILK expression in the rat heart at various intervals following injection of adenovirus containing ILK and humanized recombinant green fluorescent protein (hrGFP), from 3 to 35 days. We found that ILK and hrGFP protein expression rose progressively between 3–14 days, then declined gradually and tapered off by 35 days. In the present study, therefore, we examined ILK expression solely at 4 weeks following injection of this vector (adeno-ILK) or of the corresponding null adenovirus (adeno-null) into the left ventricle of rats. Using western blotting, we confirmed that ILK expression was elevated in the left ventricular tissue of adeno-ILK compared with adeno-null rats (Fig. 1A). The former group also exhibited an increase in the ratio of phospho-Akt to total Akt as compared with the latter group (Fig. 1B), which reflects the increase in ILK activity since Akt is a downstream target of ILK. This confirmed successful transfection and overexpression of ILK, as well as an increase in its kinase activity, in the hearts of adeno-ILK rats.

### 4.2 ILK gene therapy improves survival, cardiac function and morphology in rats with doxorubicin-induced cardiomyopathy

Following doxorubicin administration, rats began to die from day 35 (5 weeks) onwards. At day 35, animals were injected with adeno-ILK or adeno-null. In both groups, deaths occurred to similar degrees until day 43 (day 8 following adenoviral vector delivery), whereas they diverged from that point until the end of the study (day 63 following doxorubicin administration or day 28 following adenoviral vector delivery): in adeno-ILK rats, cumulative survival plateaued at 60% from day 58 (or day 23 following adenoviral vector delivery) onwards, whereas it continued to decrease to a plateau of 35% in adeno-null rats which was attained at day 59 (day 24 following adenoviral vector delivery) until the end of the study (Fig. 1C). No deaths occurred in the parallel group of rats given saline only (instead of doxorubicin). Animals not surviving to 9 weeks showed gross pathological changes consistent with cardiac failure: hepatic congestion, pleural effusions, ascites. No animal treated with ILK showed evidence of neoplasia in the liver, kidney or spleen up to the 9 week time point.

**Figure 2:**
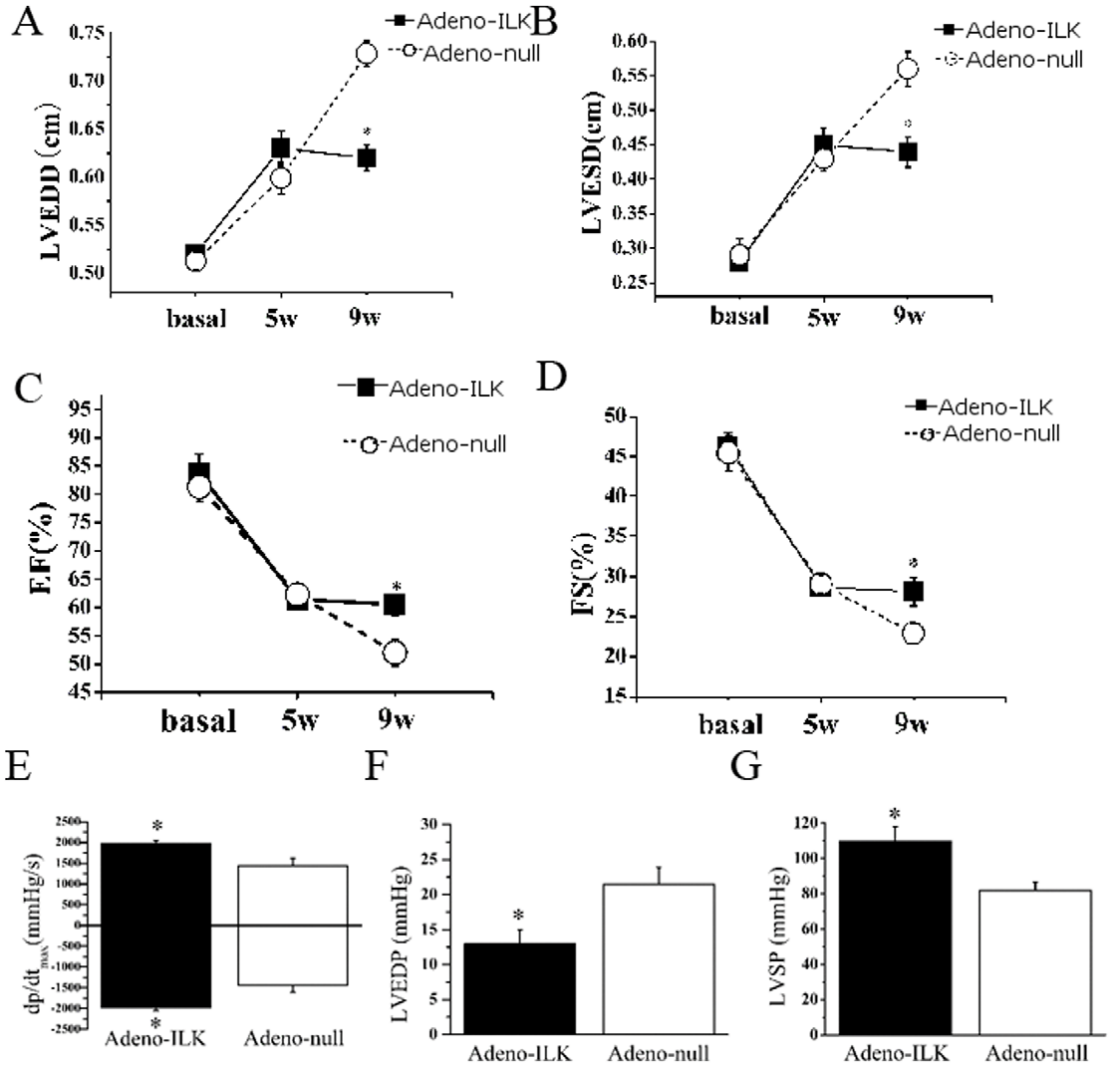
Cardiac ILK gene therapy improves cardiac function and hemodynamics in doxorubicin-induced heart failure. Shown are left ventricular end-diastolic diameter (LVEDD, A), left ventricular end-diastolic diameter (LVESD, B), ejection fraction (EF, C), fractional shortening (FS, D), ±dP/dt_max_ (E), left ventricular end-diastolic pressure (LVEDP, F) and left ventricular systolic pressure (LVSP, G). n = 20 at 5 weeks and n = 12 at 9 weeks in the adeno-ILK group, whilst n = 20 at 5 weeks and n =  7 at 9 weeks in the adeno-null group. *P<0.05 versus adeno-null group.

Systolic function, as evaluated from ejection fraction and % fractional shortening on echocardiography, deteriorated from baseline over the 5 weeks following initiation of doxorubicin treatment, and this was accompanied by an increase in left ventricular end-systolic and end-diastolic dimensions. In the surviving animals who received adeno-ILK at 5 weeks, cardiac function and dimensions at week 9 were not different from those at week 5, whereas in the adeno-null animals all of these parameters continued to deteriorate (Fig. 2 A–D). In line with these findings, we found on hemodynamic analysis that, four weeks after the adenoviral injection, there was an increase in left ventricular systolic pressure and a decrease in left ventricular end-diastolic pressure in the adeno-ILK compared with the adeno-null animals; and the adeno-ILK group also showed a greater maximum +dP/dt and a lower maximum −dP/dt as compared with the adeno-null group (Fig. 2 E–G).

**Figure 3:**
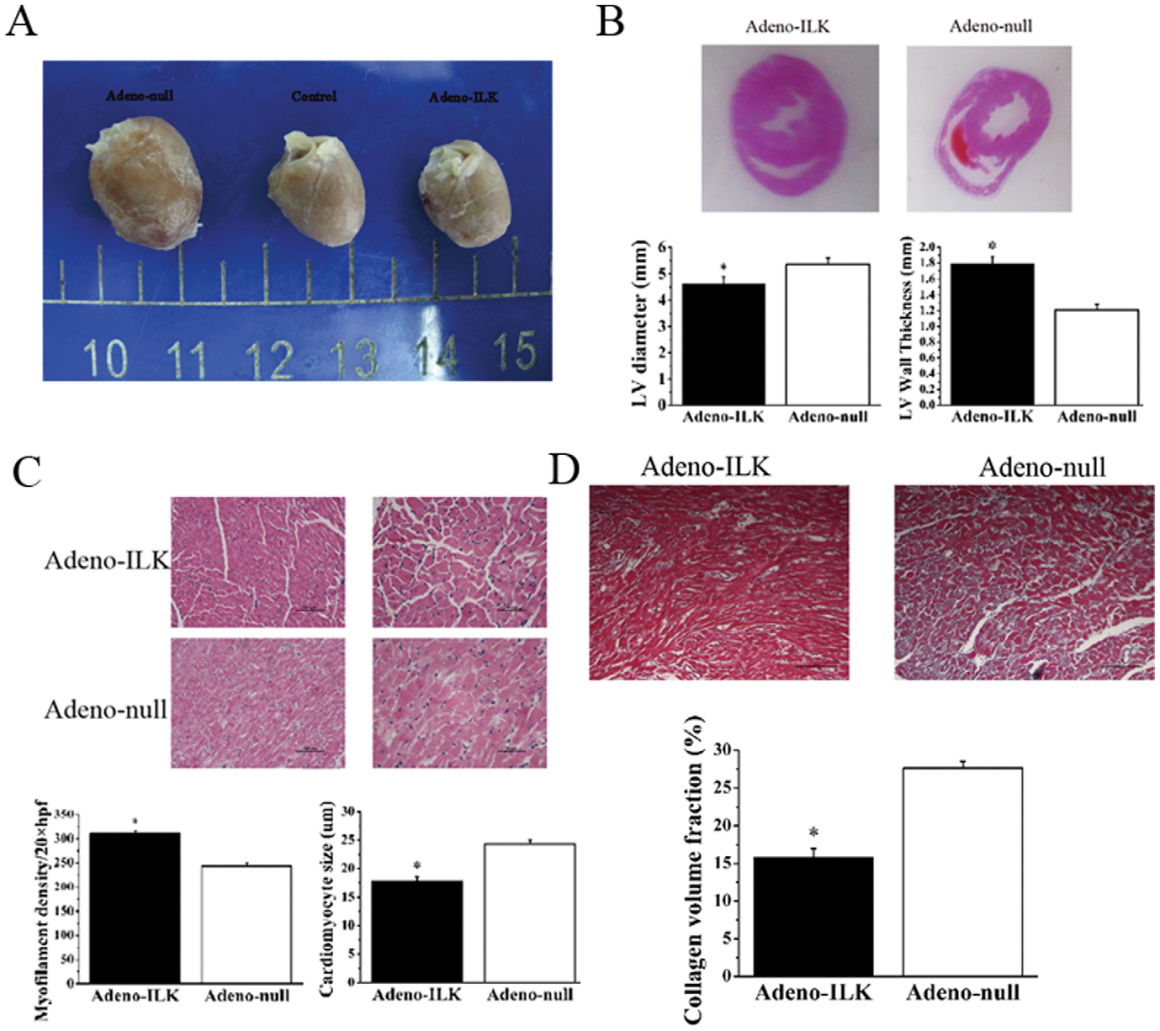
ILK gene therapy prevents left ventricular dilation, remodeling and interstitial fibrosis in doxorubicin-induced heart failure. A, Specimens showing effect of ILK treatment on heart size and shape following doxorubicin. B, Hematoxylin-eosin staining of myocardial sections 4 weeks after adeno-ILK or adeno-null treatment, with analysis of cardiac remodeling by quantification of left ventricular (LV) diameter and wall thickness. n = 6 per group. C, Myofilament density and cardiomyocyte size in adeno-ILK and adeno-null groups. n = 6 per group. Scale bars: 100 µm (left panels, 200×), 50 µm (right panels, 400×). D, Masson's trichrome staining showing infiltration by inflammatory cells and myocardial fibrosis in adeno-ILK and adeno-null animals. n = 6 per group. Scale bars: 5 mm. * *P*<0.05 vs. adeno-null.

Following sacrifice and dissection, the hearts of adeno-null rats were found macroscopically to be larger and more spherical than those of both adeno-ILK and control (saline-treated) rats (Fig. 3A). Preservation of left ventricle cavity size and wall thickness was demonstrated by decreased left ventricular diameter combined with increased wall thickness in the adeno-ILK as compared to the adeno-null group, reflecting attenuation of left ventricular dilation and global remodeling by ILK treatment (Fig. 3B). Microscopically, myofilament density was increased, and both cardiomyocyte size and interstitial fibrosis (as determined from collagen volume fraction on Masson's trichrome staining) were decreased, in adeno-ILK as compared with adeno-null rats (Fig. 3 C–D).

### 4.3 ILK gene therapy inhibits apoptosis and increases proliferation of left ventricular cardiomyocytes in rats with doxorubicin-induced cardiomyopathy

**Figure 4:**
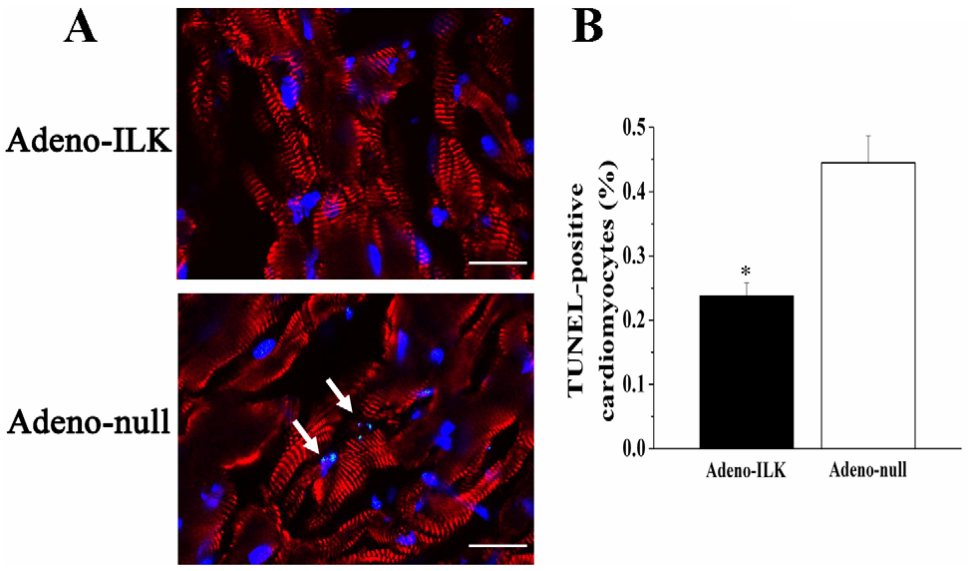
Cardiac ILK gene therapy protects left ventricular cardiomyocytes from apoptosis in doxorubicin-induced heart failure. A, Triple-staining with TUNEL (green, arrows), anti-α-sarcomeric actin antibody (red) and DAPI (blue) in left ventricle. B, Quantification of TUNEL-positive cardiomyocytes in left ventricle from adeno-ILK and adeno-null treated rats. n = 6 per group. *P<0.05 versus adeno-null group. Scale bars: 20 µm.

**Figure 5:**
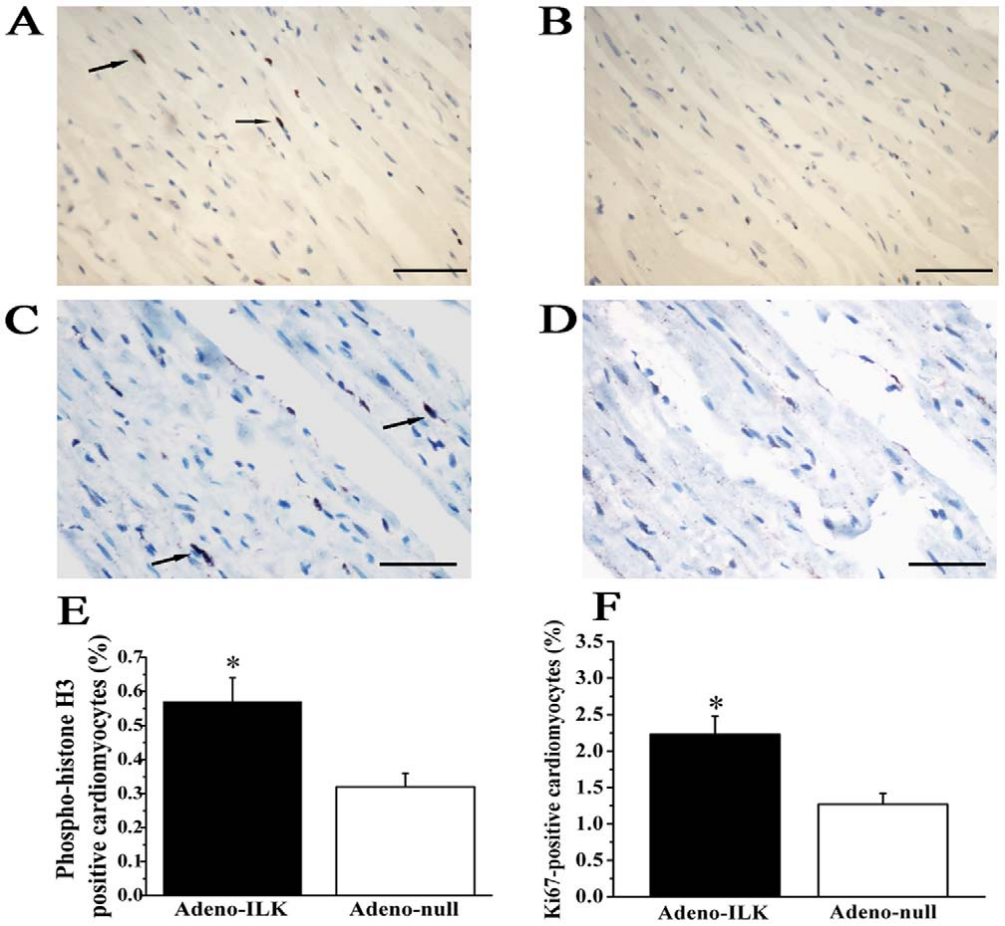
Cardiac ILK gene therapy increases left ventricular cardiomyocyte proliferation in doxorubicin-induced heart failure. A–B, Phosphohistone-H3 staining for mitotic analysis (brown, arrows), in adeno-ILK (A) and adeno-null (B) treated rats. C-D, Ki-67 staining showing more positive cells (brown, arrows) in adeno-ILK tissue sections (C) than in adeno-null sections (D). E–F, Accumulated results showing phosphohistone-H3 (E) or Ki-67 (F) positive cardiomyocytes in adeno-ILK and adeno-null groups. n = 6 per group. *P<0.05 versus adeno-null group. Scale bars: 100 µm.

TUNEL analysis and phosphohistone-H3 expression together with Ki-67 expression were performed to quantify cardiomyocyte apoptosis and proliferation respectively. We found that the percentage of TUNEL-positive cardiomyocytes was decreased in the adeno-ILK compared to the adeno-null group (Fig. 4). On the other hand, phosphohistone-H3 or Ki-67 expression localized to cardiomyocytes were both found to increase in the adeno-ILK group relative to the adeno-null group, suggesting that ILK treatment increases cardiomyocyte proliferation (Fig. 5). To investigate whether the proliferating cells were of stem cell origin, we examined Sca-1 positive cells but found no difference in this between the adeno-null and adeno-ILK groups (data not shown).

### 4.4 Doxorubicin treatment increases circulating markers of inflammation and oxidative stress, and these are inhibited by cardiac ILK gene therapy

**Figure 6:**
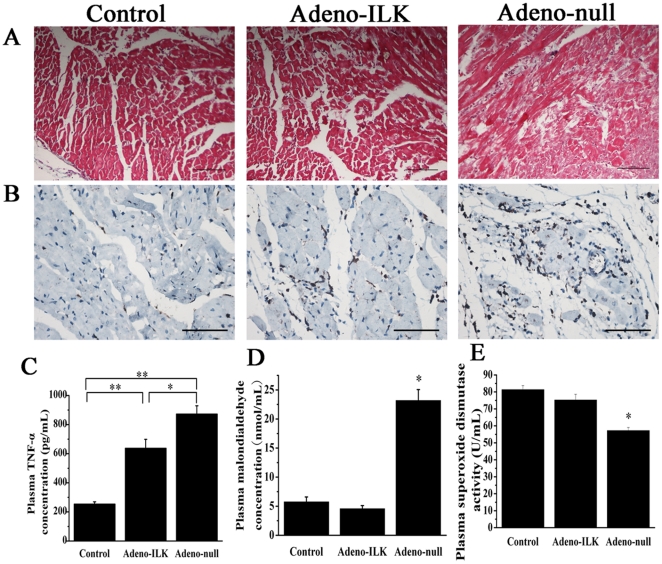
Cardiac ILK gene therapy prevents inflammation and cardiomyocyte degeneration, and improves markers of systemic inflammation and oxidative stress, in doxorubicin-induced heart failure. A–B, Hematoxylin-eosin (A) and CD45 staining (brown) (B) of myocardium 4 weeks following adeno-ILK or adeno-null treatment, showing effects on cardiomyocyte degeneration (myofibrillar derangement and disruption) and inflammatory cell infiltration. C–E, Plasma tumor necrosis factor-α (TNF-α) concentration (C), malondialdehyde concentration (D) and superoxide dismutase activity (E), in control (saline-treated) as well as doxorubicin-treated rats receiving either adeno-ILK or adeno-null. n = 6 per group. *, ** *P*<0.05 and <0.01 respectively. Scale bars: 100 µm.

Inflammatory cell infiltration and cardiomyocyte degeneration, as evidenced by CD45 and hematoxylin-eosin staining, were ameliorated by ILK treatment (Fig. 6 A–B). Plasma tumor necrosis factor-α (TNF-α) concentration, a marker of systemic inflammation, was higher in doxorubicin- than saline-treated rats, and this increase was attenuated in part by cardiac ILK overexpression (Fig. 6C). Doxorubicin also gave rise to an increase in oxidative stress, as evidenced by a rise in plasma malondialdehyde (MDA) concentration and a decrease in plasma superoxide dismutase (SOD) activity compared with the saline-treated rats; ILK overexpression abrogated the increase in plasma MDA as well as the decrease in plasma SOD activity, compared with the adeno-null controls (Fig. 6 D–E).

### 4.5 Doxorubicin treatment causes cardiac myofibril degeneration and autophagic vacuole formation, effects which are inhibited by ILK gene therapy

**Figure 7:**
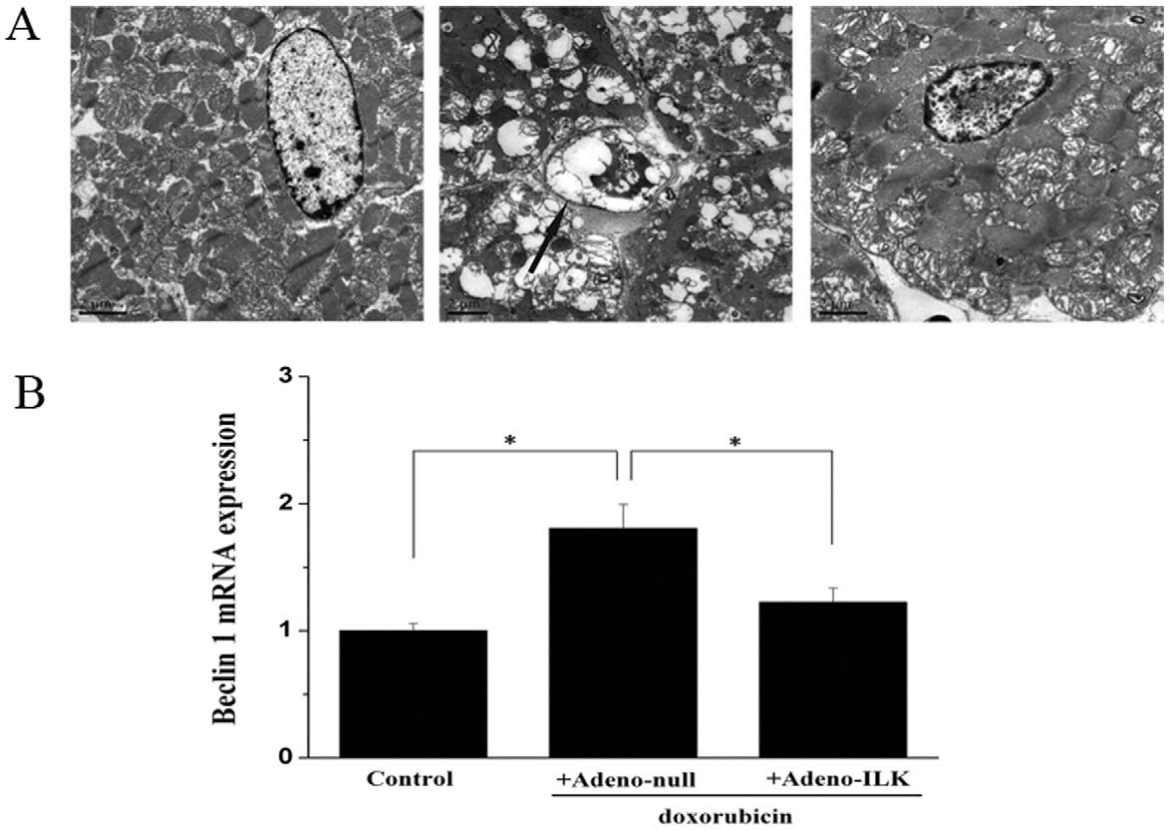
Cardiac ILK gene therapy inhibits doxorubicin-induced autophagy. A, Electron micrographs showing autophagic vacuole (arrow) formation in cardiomyocyte of a doxorubicin-treated rat administered adeno-null, and lack of this in another rat administered adeno-ILK and in a control (saline-treated) rat. Scale bar, 2 µm. B, Effect of doxorubicin treatment on cardiac beclin 1 mRNA expression, and the effect of ILK treatment on this. Beclin 1 mRNA expression levels were normalized first to GAPDH and then displayed relative to levels in the control group using the2^−ΔΔCt^ method. n = 6 per group. *P<0.05.

Compared with the hearts of rats treated with saline, doxorubicin gave rise to accumulation of autophagic vacuoles, altered structural integrity and extensive degeneration of myofibrils. The hearts from adeno-ILK animals displayed a decrease in the number of autophagic vacuoles and a finer mitochondrial ultrastructure (Fig. 7A).

Since the formation of the autophagasome is critically dependent on beclin 1, we also quantitated beclin 1 mRNA expression by real-time PCR. We found this to be increased 1.8-fold in the hearts of doxorubicin-treated rats administered adeno-null, as compared to control (saline-treated) rats. By contrast, this increase was abrogated in doxorubicin-treated rats given adeno-ILK (Fig. 7B).

### 4.6 ILK attenuates doxorubicin-induced apoptosis, and promotes proliferation, of cultured neonatal rat cardiomyocytes

**Figure 8:**
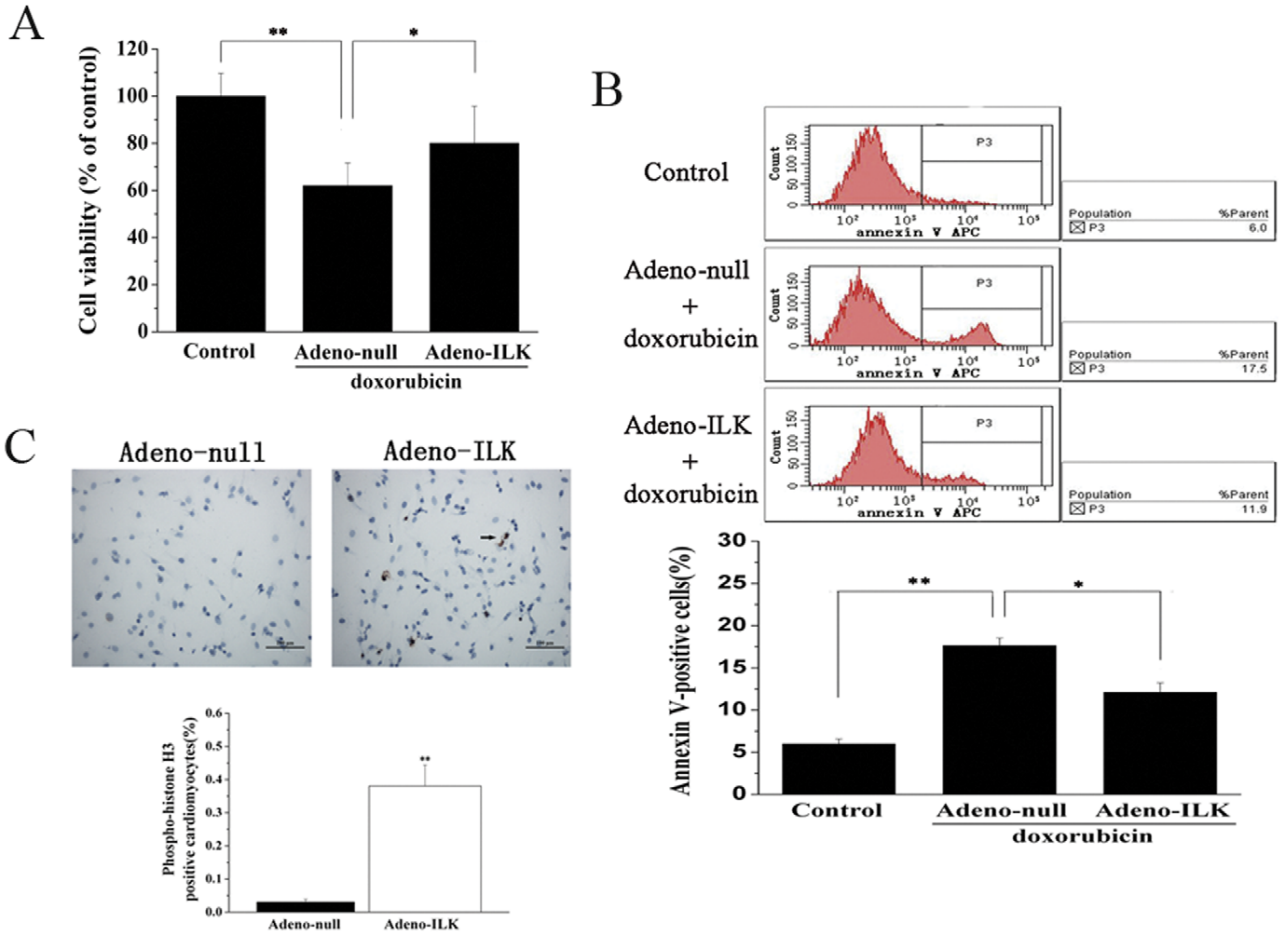
Adeno-ILK prevents apoptosis, and promotes proliferation, of cultured neonatal rat cardiomyocytes treated with doxorubicin. A, Effect of doxorubicin on cell viability, as assessed by MTT assay, and the effect of ILK on this. n = 6 per group. *, **P<0.05 and <0.01 respectively. B, Effect of doxorubicin on cardiomyocyte apoptosis, as assessed by annexin V expression (determined by flow cytometry), and the effect of ILK on this. Necrotic cells were excluded from the analysis by gating on PI-negative cells. n = 6 per group. *, **P<0.05 and <0.01 respectively. C, Effect of ILK on proliferation of neonatal rat cardiomyocytes treated with doxorubicin, as assessed by phosphohistone-H3 staining (brown); this showed more positive nuclei (arrow) in the adeno-ILK group than the adeno-null group. Graph shows accumulated results of n = 6 experiments. **P<0.01 versus adeno-null group. Scale bar, 100 µm.

Neonatal rat cardiomyocytes were isolated and cultured; we found that >90% of the cells in culture exhibited immunocytochemical staining for alpha sarcomeric actin in the cytoplasm, confirming their nature as cardiomyocytes as well as their high degree of purity. Incubation of cells with 1 µmol/L doxorubicin for 24 h resulted in decreased cell viability, as measured by 3-(4,5-dimethylthiazol-2-yl)-2,5-diphenyltetrazolium bromide (MTT) assay; cardiomyocytes transfected with adeno-ILK prior to addition of doxorubicin exhibited enhanced cell viability relative to adeno-null transfected cells (Fig. 8A).

Cardiomyocyte apoptosis was increased in the presence of doxorubicin. As shown in Fig. 8B, the early apoptotic marker annexin V was increased in adeno-null transfected cardiomyocytes, but this change was attenuated in adeno-ILK transfected cells, in the presence of doxorubicin. Furthermore, a marked increase in phosphohistone H3-positive cells was found in the adeno-ILK group as compared to the adeno-null group (Fig. 8C), indicating an increase in their rate of proliferation in the former group compared to the latter.

## Discussion

The present study provides evidence of benefit for ILK treatment on the progression of heart failure caused by doxorubicin-induced cardiomyopathy, a non-ischemic cardiomyopathy whose pathophysiological features closely resemble those of dilated cardiomyopathy in humans. Treatment with adeno-ILK was started 5 weeks after the first doxorubicin injection, so that the rescue effects could be assessed once cardiac function had already been compromised. In our model, the cardiomyopathy was severe, as evidenced by a clear increase in mortality, advanced signs of cardiac dysfunction, and significant histopathologic changes in the doxorubicin -treated animals. We found that ILK administration improved the prognosis of dilated cardiomyopathy in terms of survival, with associated marked improvements in both functional and structural parameters after ILK treatment: enhanced cardiac function, relatively preserved left ventricular diameter and wall thickness, attenuated interstitial fibrosis, decreased cardiomyocyte apoptosis, increased cardiomyocyte proliferation, decreased inflammatory cell infiltration, reduced oxidative stress and diminished autophagic vacuole accumulation.

As a critical component of the cardiac mechano-sensor/transduction mechanism, ILK functions as a scaffold linking cell-surface integrins to intracellular signaling pathways and the actin cytoskeleton. ILK is activated by mechanical stretch and growth factors, and thereby affects important signaling pathways within the cardiomyocyte which induce cell survival, promote angiogenesis and increase cardiomyocyte contractility [8]. Targeted knock-out of ILK in the murine heart results in disaggregation of cardiomyocytes and spontaneous dilated cardiomyopathy, suggesting that ILK plays an important part in protecting the heart from cardiomyopathy and heart failure. ILK increases cardiomyocyte hypertrophy and contractility by upregulating atrial natriuretic peptide and VEGF expression [8], which also both exert protective effects on cardiac function [19]. Previous studies have shown that ILK can induce angiogenesis, cell growth, proliferation, survival and differentiation in non-cardiac cells [10], whilst we have previously shown these same effects on cardiomyocytes following ILK treatment in the context of myocardial infarction [13].

Myocardial apoptosis is a major determinant of reduction in cardiac function [20]. In a model of acute myocardial infarction, we have previously demonstrated that ILK treatment reduces cardiomyocyte apoptosis both in the border and more remote zones [13]. In the present study, we have similarly found that ILK treatment improves cardiac function secondary to reduced apoptosis in doxorubicin-induced cardiomyopathy. Since ILK overexpression can promote activation of Akt [12], which in turn can inhibit cardiomyocyte apoptosis and improve cardiac contractility [21], we postulate that ILK exerts these beneficial effects through increasing the phosphorylation and hence activation of Akt.

Irreversible cardiomyocyte loss, coupled with limited regenerative capacity and ability to differentiate, following a variety of environmental stresses and external injuries are believed to be important factors leading to the progression of cardiac dysfunction. Previous studies have shown that ILK overexpression can promote cardiac stem cell amplification in vitro [22] as well as induce cyclin D2 expression [23], which can promote cardiomyocyte regeneration in injured hearts [24]. In the present study, we observed increased cardiomyocyte proliferation in the left ventricle in response to ILK treatment, and this is likely also to contribute to the observed amelioration of cardiac function. Our experiments provided no evidence that this increase in cardiomyocyte proliferation in response to ILK was explained by stem cell differentiation, and this is consistent with previous studies [25].

We observed that doxorubicin induced myocardial fibrosis and inflammatory cell infiltration, and that ILK treatment prevented both of these processes. It is already established that elevation in inflammatory mediators in the heart is associated with cardiac dysfunction [26], but it has been contentious as to whether TNF-α mediates inflammation and fibrosis in doxorubicin-induced cardiomyopathy and the failing heart [27]–[30]. We here report decreased plasma TNF-α concentration in adeno-ILK compared to adeno-null treated rats, which is in line with the observed reduction in inflammatory cell infiltration in the adeno-ILK group; the underlying mechanism, however, requires further investigation.

Many mechanisms have been proposed to explain doxorubicin-mediated cardiotoxicity, among which the generation of reactive oxygen species (ROS) and calcium overload are considered perhaps the most important [31]–[33]. ROS are also considered important in the pathophysiology and progression of idiopathic dilated cardiomyopathy [34]. ROS cause lipid peroxidation and resultant generation of degradation products such as MDA. On the other hand, the activities of antioxidant systems such as SOD decrease under conditions of oxidative stress. In the present study, an increase in MDA and decrease in SOD activity in the plasma were observed in doxorubicin-treated rats, which were restored by ILK treatment, indicating that cardiac ILK gene therapy reverses ROS overproduction and subsequent oxidative stress.

Autophagy is considered physiologically important for the maintenance of normal cardiovascular morphology and function, whilst excessive autophagy by various factors contributes to the induction and exacerbation of several types of cardiomyopathy [35], [36]. We found that doxorubicin-treated rats receiving adeno-ILK displayed a decreased number of autophagic vacuoles compared to those receiving adeno-null. In line with this finding, we also observed a reduction in beclin 1 (a Bcl-2-interacting protein) expression in the adeno-ILK group. Our findings suggest that cardiac ILK treatment can reduce autophagic activity, and this may contribute to delaying, or even reversing, the progression of heart failure.

In the present study, we found both increased expression and kinase activity of ILK in the heart after adeno-ILK delivery. We evaluated the safety of ILK-treatment at four weeks after adenoviral delivery, especially in view of its propensity to induce angiogenesis and proliferation, but no neoplasia was detected in the liver, kidney or spleen of treated rats. Our study therefore suggests that ILK gene therapy beneficially affects left ventricular structure and function in doxorubicin-induced cardiomyopathy, as well as improving survival. Its potential usefulness in dilated cardiomyopathy in humans remains to be determined.
